# A gist on an obscure neoplasm in Ghana: gastrointestinal stromal tumours

**DOI:** 10.1186/s13104-023-06593-8

**Published:** 2023-11-06

**Authors:** Joseph Yorke, Samuel Gyasi Brenu, Ronald Awoonor-Williams, Stephen Tabiri, Anwar Sadat Seidu, Francis Akwaw Yamoah, Joseph Akpaloo, Edmund Muonir Der, Ernest Adjei, Isaac Okyere, Kenneth Kelechi Ihekanandu, Ernest Bawuah Osei Bonsu, Ishmael Kyei, Samuel Mensah, Michael Ofoe Adinku, Dennis Afful Yorke, Akwasi Opoku Agyapong, Francis Somiah-Kwaw Aitpillah, Martin Kofi Agyei, Nana Akosua Oppong-Nkrumah, Kwasi Dadzie Annan, Theodora-Ann Fremponma Ellis, Patrick Danso, Tonnies Abeku Buckman, Emmanuel Acheampong

**Affiliations:** 1 Department of Surgery, School of Medicine and Dentistry, Kwame Nkrumah University of Science and Technology, Kumasi, Ghana; 2 Directorate of Surgery, Komfo Anokye Teaching Hospital, Kumasi, Ghana; 3 Directorate of Trauma & Orthopaedics, Komfo Anokye Teaching Hospital, Kumasi, Ghana; 4 Department of Surgery, School of Medical Sciences, University of Development Studies, Tamale, Ghana; 5 Department of Surgery, Tamale Teaching Hospital, Tamale, Ghana; 6 Department of Pathology, Tamale Teaching Hospital, Tamale, Ghana; 7 Directorate of Pathology, Komfo Anokye Teaching Hospital, Kumasi, Ghana; 8 Department of Molecular Medicine, School of Medicine and Dentistry, Kwame Nkrumah University of Science and Technology, Kumasi, Ghana; 9 Directorate of Oncology, Komfo Anokye Teaching Hospital, Kumasi, Ghana; 10 Directorate of Internal Medicine, Komfo Anokye Teaching Hospital, Kumasi, Ghana; 11 Directorate of Emergency Medicine, Komfo Anokye Teaching Hospital, Kumasi, Ghana; 12 Department of Medical Laboratory Science, KAAF University College, Fetteh-Kakraba, Gomoa East District, Gomoa-East, Ghana

**Keywords:** Gastrointestinal stromal tumours, Partial gastrectomy, Imatinib

## Abstract

**Background:**

Gastrointestinal Stromal Tumour is a rare but potentially curable tumour of the gastrointestinal tract accounting for up to 1% of all gastrointestinal tumours. The discovery of Imatinib mesylate, a novel tyrosine kinase inhibitor has improved the chances even for unresectable, recurrent, or metastatic diseases.

**Methods:**

This study sought to document the clinical and pathological characteristics of GISTs from two tertiary hospitals in Ghana that have undergone immunohistochemistry confirmation between 2014 and 2021.

**Results:**

The median age of the subjects was 50 years with most of them (28.0%) being above 61 years. There were more females than males (64.0% vs. 36.0%). Abdominal mass and abdominal pain made up the majority of the clinical presentations. The majority of the subjects had partial gastrectomy (32.0%) which was followed by wedge resection (28.0%). Appendectomy and sleeve gastrectomy were the least performed procedures (8% each). Four of the 25 patients (16.0%) had resections of involved contiguous organs done with splenectomy being the most common procedure. The majority of GISTs were found in the stomach (68.0%) followed by the appendix (12.0%) and small bowel (12.0%). Gastrointestinal bleeding (55.8%) and abdominal pain (38.5%) were the most reported symptoms. Free resection margins were observed in 84.0% of the subjects and only 3/25 (12.0%) experienced tumour recurrence.

**Conclusion:**

GIST is a potentially curable tumour that once was obscure but currently gaining popularity. Surgical resection offers the hope of a cure for localized disease while targeted therapies is a viable option for recurrent, metastatic, or unresectable tumours.

## Introduction

Gastrointestinal Stromal Tumours (GISTs) are a common group of stromal or mesenchymal neoplasms arising from the gastrointestinal tract which account for approximately 1% of gastrointestinal tumours in adults [1]. GISTs have in the past been defined as smooth gastrointestinal muscle tumours and have frequently been misdiagnosed as leiomyomas, schwannomas, and sarcomas over the years [2]. While GIST can develop anywhere in the digestive tract, the majority originate from the stomach (50–70%) and to a lesser extent in the duodenum, jejunum, and ileum of the small intestine (20–30%). Additional sites include the large intestine (5%), and the oesophagus (2–5%) [3–5].

The incidence of GISTs has been rising over the past decades, with most published studies reporting 10 to 15 new cases per 100,000 individual years [5]. The clinical presentation of GISTs is dependent on the location and size of the primary tumour. GIST can be symptomatic in up to 80% of cases and they are typically associated with symptoms of tumour mass effects such as abdominal pain, discomfort, distension, palpable mass, anaemia, and GI haemorrhage [4, 6, 7]. The most frequent complaints documented in the literature are abdominal discomfort and gastrointestinal bleeding [8, 9]. Small-sized GISTs, on the other hand, are frequently asymptomatic and found by chance.

GISTs are suspected in imaging and endoscopic examinations and the diagnosis is confirmed by tissue acquisition with immunohistochemical staining. The aggressiveness of GISTs depends on size, mitotic index, and location. Surgical resection is the treatment modality of choice but that alone is not curative for all patients especially those with high-risk GIST [4, 6, 10, 11]. With surgically removed tumours, about 50% of patients experience local or metastatic recurrence. The most important prognostic factors for patients following surgery are the tumour size, mitotic rate, and tumour site. However, without adjuvant therapy, malignancy is still a possibility regardless of the tumour’s size or mitotic rate [12] which highlights the significance of additional therapy in the treatment of GIST [13, 14].

GIST incidence varies greatly between geographical regions, with Sub-Saharan Africa experiencing a sharp increase in incidence. However, there isn’t much information available about its occurrences and anatomical distribution in Ghana. Given that GIST is medically treatable, its initial obscurity makes early detection and treatment more challenging, which justifies the need for greater awareness of this disease. Also, this description would serve as a premise on which future characterizations of GISTs in Ghana and the continent at large could be made. It would also offer an opportunity to ascertain if similar characteristics of GISTs pertains here as there is in the developed countries. This study sought to document the clinical and pathological characteristics of GISTs from two tertiary hospitals in Ghana that have undergone immunohistochemistry confirmation.

## Materials and methods

### Study design and area

The study is a multicentre cross-sectional retrospective study of all cases of GIST in two (2) tertiary healthcare facilities in Ghana; Komfo Anokye Teaching Hospital (KATH) and Tamale Teaching Hospital (TTH). They are the second and third largest Teaching Hospitals in Ghana respectively. They serve the tertiary healthcare needs of the middle and northern belts of Ghana. The Komfo Anokye Teaching Hospital is in the Kumasi Metropolis of the Ashanti Region of Ghana with a 1200-bed capacity. Tamale Teaching Hospital, on the other hand, is in the capital city of the Northern Region of Ghana with a bed capacity of 800.

### Study population and subject selection

The population constituted patients diagnosed with GIST in the two hospitals between January 2014 and June 2022 who underwent surgeries in both hospitals. Patients diagnosed with GIST within the stated period who had more than 30% of data loss were excluded.

### Data collection

The archived data of the Departments of Surgery of both hospitals were searched looking for diagnoses of GIST made within the stated period of the study. Each patient record was then perused, and the required data points were extracted onto an Excel sheet. The data variables recorded include patient demographics, clinical presentation, hemogram findings, endoscopic and radiologic findings, preoperative biopsy report, intraoperative location of the tumour, presence of associated tumour-related complications, presence of surrounding organ involvement, liver metastasis, type of resection done, histopathologic analysis, immunohistochemistry findings and the presence of recurrence. A total of 8 cases were retrieved from KATH and 17 cases from TTH. Both sets of data were synchronized onto one Excel sheet for analysis.

### Surgical technique

All patients were evaluated clinically with detailed history and clinical examination. All surgeries were approached via a midline laparotomy incision to the peritoneal cavity. The presence of ascites, peritoneal seedlings, intraperitoneal haemorrhage, and liver metastases were looked out for. The tumours were identified, and respective resections were done based on their locality. Partial gastrectomy with Billroth II reconstruction was required for large gastric tumours located in the antral region while wedge resections were carried out for small tumours in the gastric body. An open-sleeve gastrectomy was done for one of the patients who had the tumour occupying almost two-thirds of the fundus and body of the stomach. The small bowel tumours had small bowel resection with end-to-end anastomosis done. A right hemicolectomy with ileo-colic anastomosis was required for the right colonic and mesenteric tumours. Involved contiguous organ resections such as splenectomy, transverse colectomy with colo-colic anastomosis, and subtotal abdominal hysterectomy were performed. Restoration of gut continuity was done with staples or hand suturing using vicryl 2/0. Abdomen was irrigated with distilled water and closed in layers. All patients were followed up on an out-patient basis at 2 weeks, 1 month, 3 months, and 6 months post-op.

### Immunohistochemistry

**Fig. 1:**
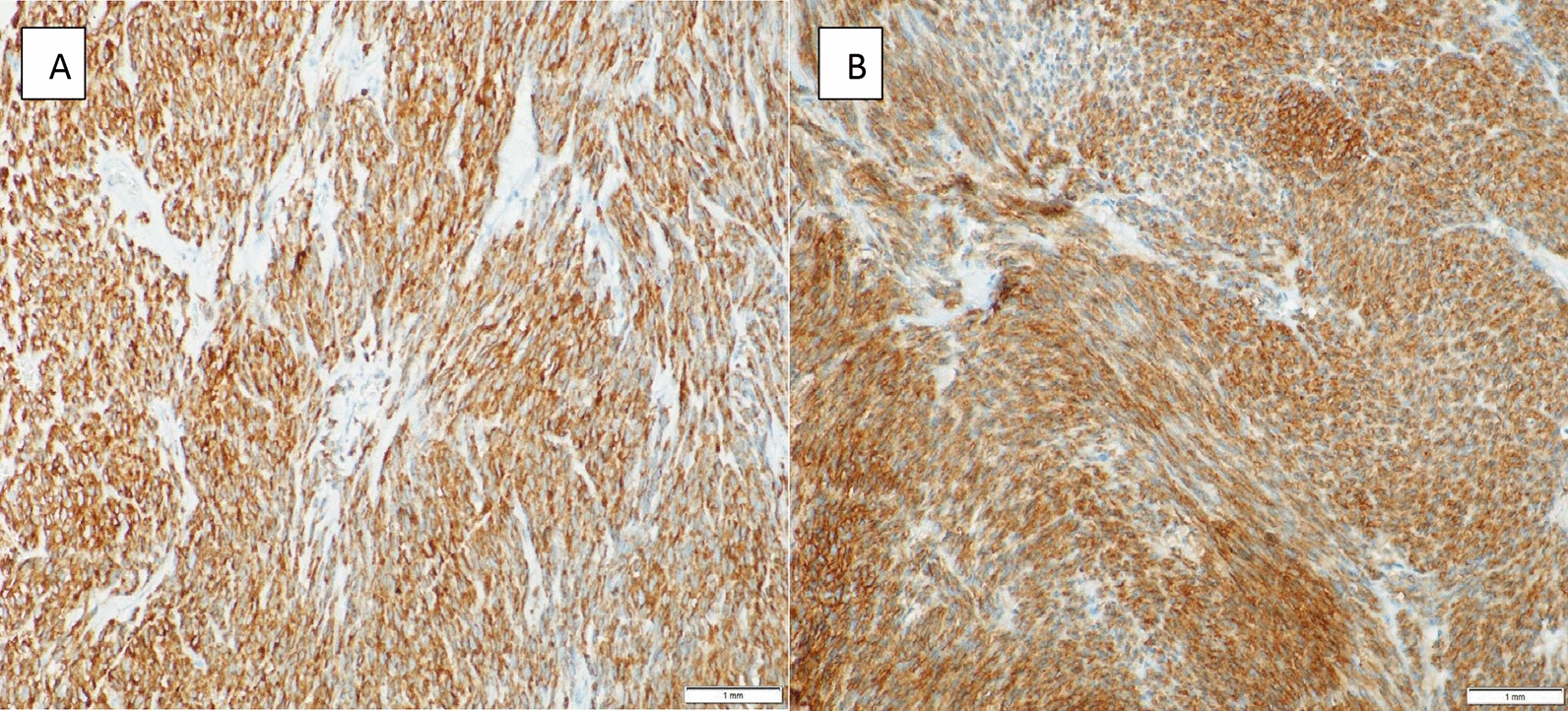
Immunohistochemistry staining with CD117 (C-Kit) in **A** and DOG 1 in **B** at ×300 magnification

All cases were first evaluated by standard H&E-stained slides. The morphological features were recorded, and the histologic diagnosis of GIST was made. All the cases were further evaluated with Immunohistochemistry staining. The standard stains used were DOG1, CD117, SM-actin, S100, AE1-AE3, and Vimentin. Cases were classified as GIST confirmed if they were either positive for DOG1 or CD117 or both. Figure 1 below depicts immunohistochemistry staining with CD117 (A) and DOG1 (B) of one patient captured at 300× magnification. Only one case from KATH was negative for both markers and next-generation sequencing was requested.

### Data analysis

Archived data was de-identified and data processing was done using Microsoft Excel 2019. Categorical data were presented as frequencies and percentages. Parametric and non-parametric continuous data were presented as means ± SD and medians (IQR), respectively. All figures were developed using ggplot package R software version 4.2.1 (https://www.r-project.org/).

## Results

**Fig. 2:**
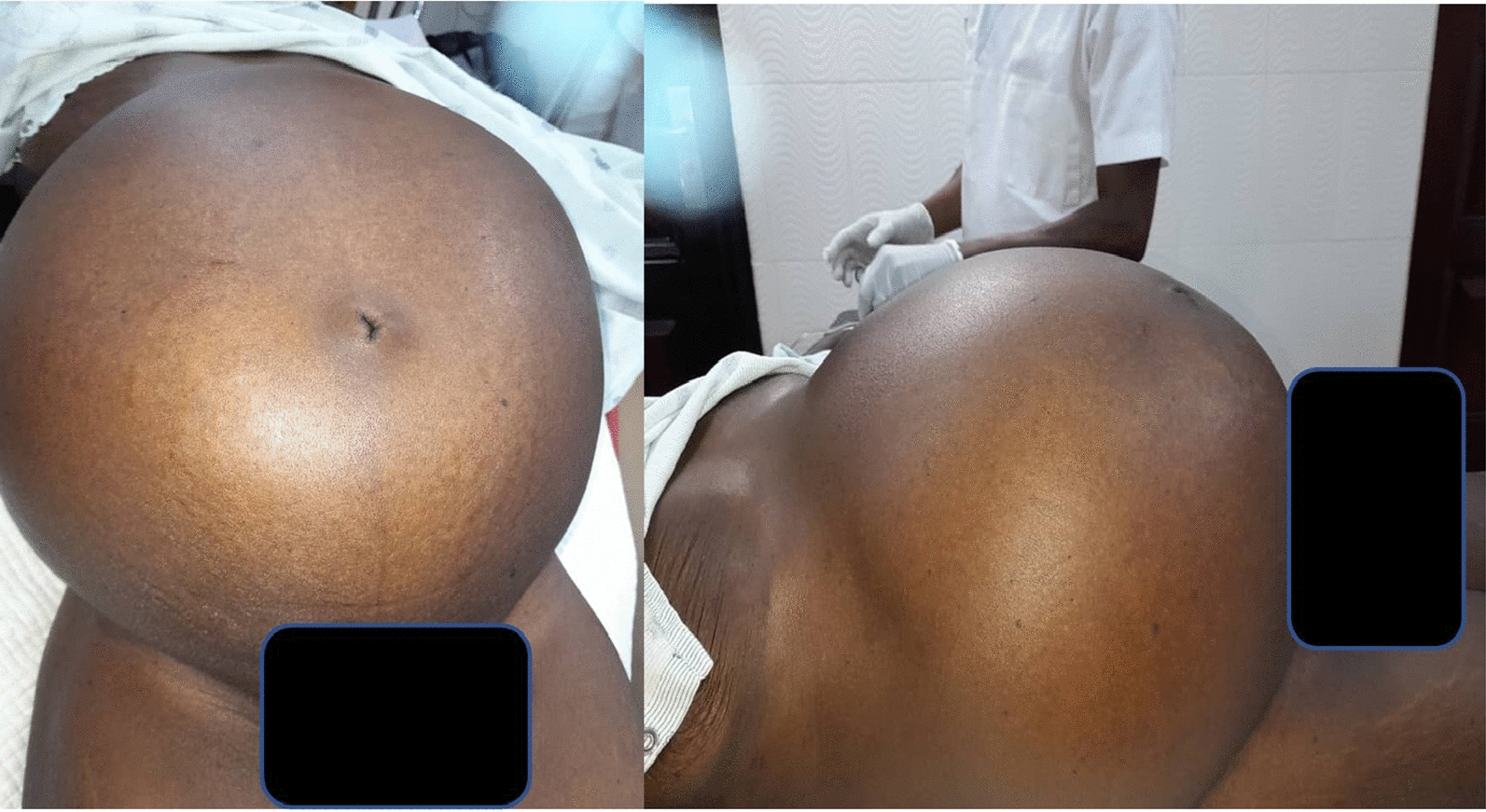
Gross abdominal distension due to massive ascites of one patient

Table 1 shows the clinico-demographical characteristics of the study population. A total of 25 patients with complete medical records were retrieved from the archives of both institutions within the study period. The median age of the subjects was 50 years. Most of them (28.0%) were above the age of 61, followed by equal distribution falling between 31 and 40 (24.0%) and 51–60 (24.0%) years respectively. The median haemoglobin level of the participants was 5.5 g/dL. There were more females (64.0%) than males (36.0%). Most subjects had an abdominal mass (60.0%) and 12 participants (48.0%) reported having abdominal pains. Figure 2 shows a pictorial view of one patient who presented with gross abdominal distension due to a large gastric tumour as well as massive ascites.

Three subjects of the total participants had lower GI bleeding (12.0%) while seven of the subjects had upper GI bleeding (28.0%).

**Table 1:** Clinico-demographical features: age, gender, and clinical presentation

Variables	Frequency (n = 25)	Percentage (%)
Age (years), median (IQR)	50 (36–76)	
Age group (years)		
21–30	3	12
31–40	6	24
41–50	3	12
51–60	6	24
≥ 61	7	28
HB (g/dL) on admission, median (IQR)	5.5 (4.9–7.3)	
Gender		
Male	9	36
Female	16	64
Abdominal mass		
No	10	40
Yes	15	60
Abdominal pain		
No	13	52
Yes	12	48
Upper GI bleeding		
No	18	72
Yes	7	28
Lower GI bleeding		
No	22	88
Yes	3	12

*IQR* interquartile range, *GI* gastrointestinal

Table 2 details the diagnostic approaches and pathological features of the subjects. More than half of the subjects had computerised tomography (CT) scans (68.0%) and pre-operative biopsy (52%). The stomach represented the most clinically presented tumour location (68%) followed by the appendix and small bowel in equal proportion (12.0% each). Intraluminal bleeding, intraperitoneal bleeding, and intestinal obstruction were reported complications seen in 32.0%, 12.0%, and 12.0% of the subjects respectively. Metastatic disease involving the peritoneum and liver were observed in 20% and 8% of the subjects respectively. Six of the total subjects (24.0%) had adjacent organ involvement. All but one case was histopathologically diagnosed as extra-intestinal GIST (4.0%) and most of the cases (66.0%) were classified as high-risk based on the histopathological risk criteria scores.

**Table 2:** Frequency distribution of surgical characteristics

Variables	Frequency (n = 25)	Percentages (%)
Pre-operative biopsy		
No	12	48
Yes	13	52
Tumour location		
Appendix	3	12
Colon	1	4
Mesentery	1	4
Small bowel	3	12
Stomach	17	68
Intraluminal bleeding		
No	17	68
Yes	8	32
Intraperitoneal bleeding		
No	22	88
Yes	3	12
Intestinal obstruction		
No	22	88
Yes	3	12
Peritoneal metastasis		
No	20	80
Yes	5	20
Liver metastasis		
No	23	92
Yes	2	8
Adjacent organ involvement		
None	19	76
Yes	6	24
Histopathological diagnosis		
GIST	24	96
Extra interstitial GIST	1	4
Histopathological risk category		
Low	11	44
High	14	56

*CT scan* computerised tomography scan, *GIST* gastrointestinal stromal tumours

**Fig. 3:**
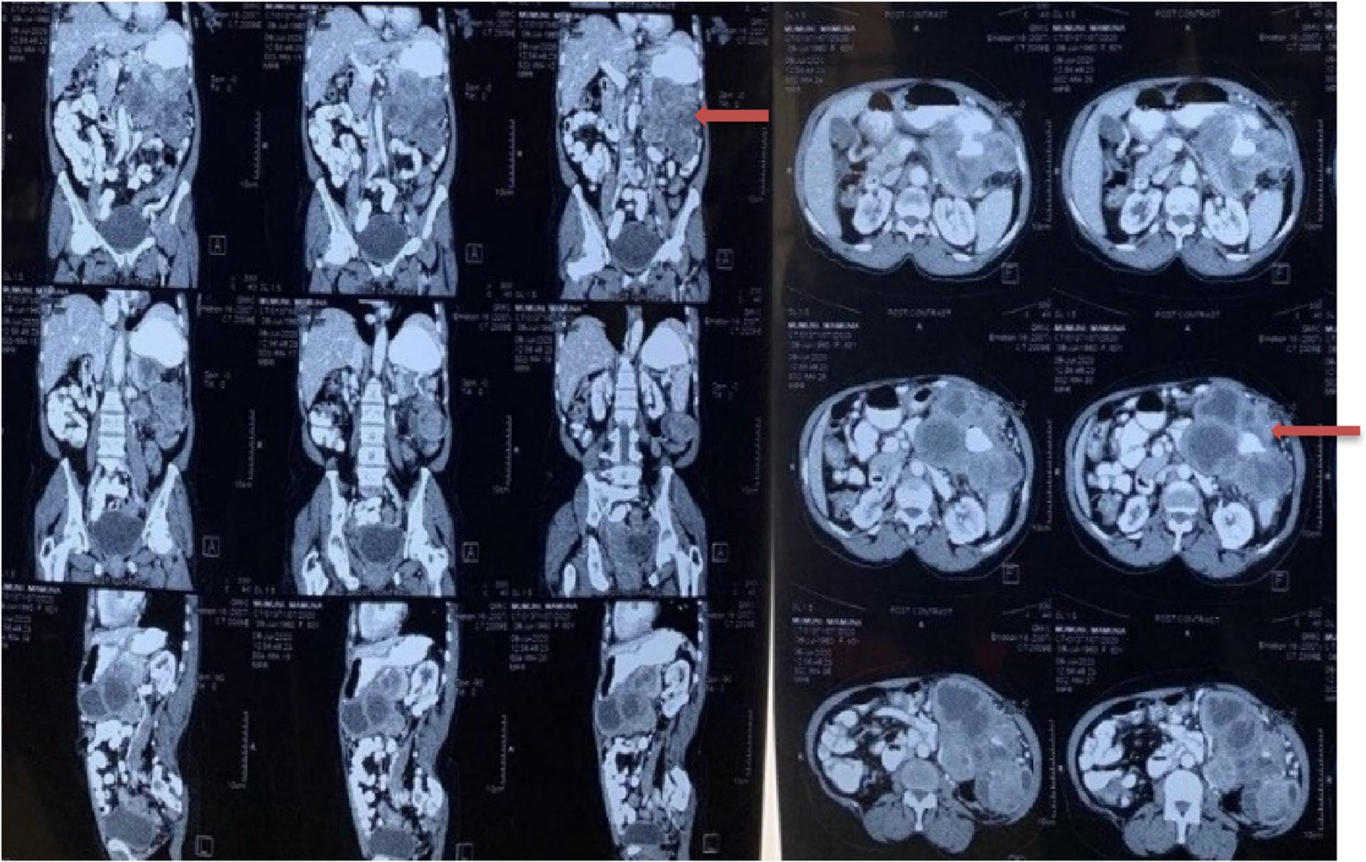
Abdominal CT scan of one patient showing mass (red arrow) originating from the stomach and invading spleen, pancreas, and small bowel

**Fig. 4:**
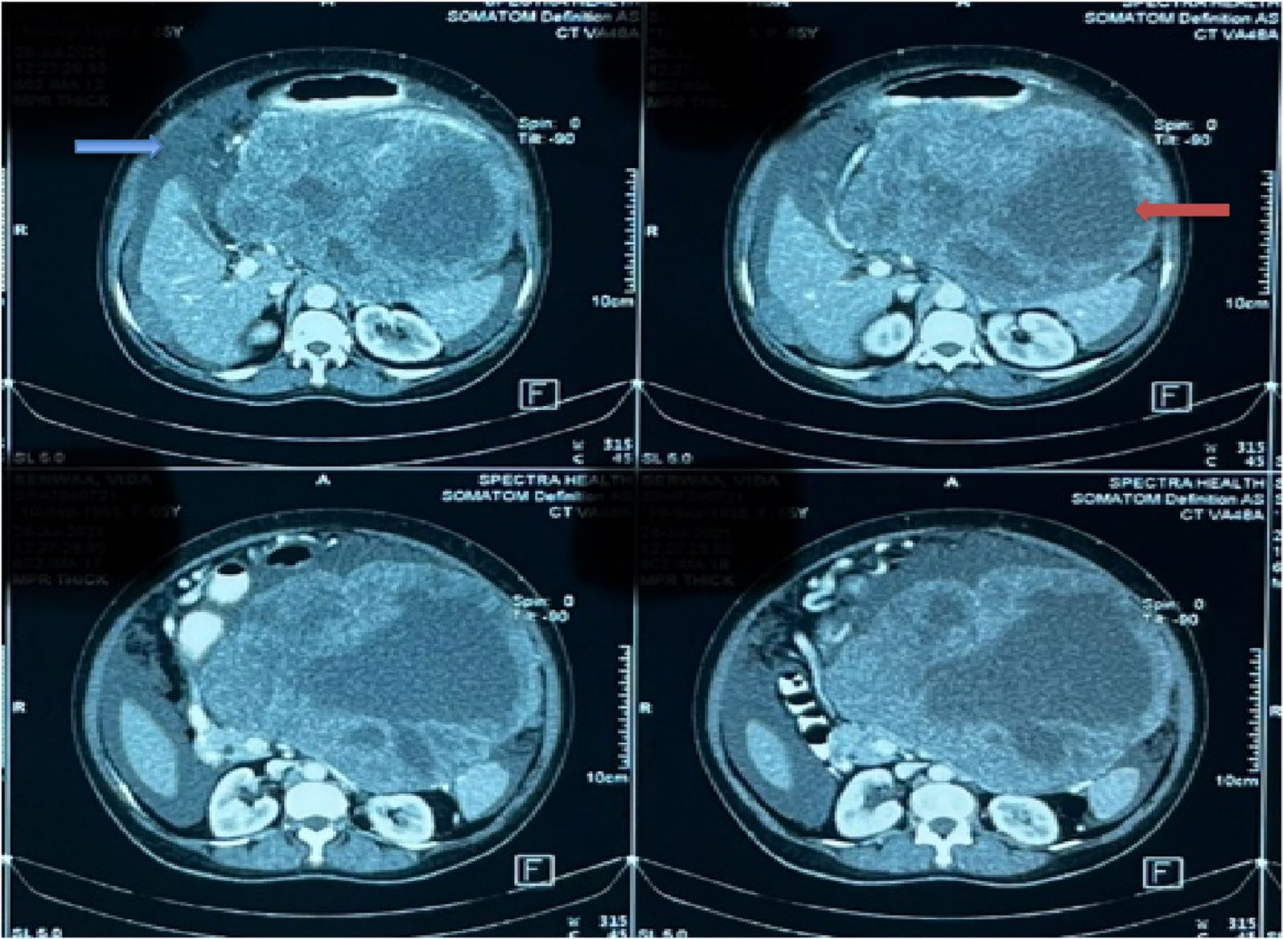
Abdominal CT scan showing heterogenous gastric tumour (red arrow) with ascites (blue arrow)

Figure 3 shows the CT scan of one patient depicting a heterogenous and exophytic mass emanating from the stomach and involving the transverse colon, pancreas, and spleen. There are no metastatic hepatic lesions evident. Figure 4 also shows a CT scan of another patient showing a large complex heterogenous mass originating from the stomach in contiguity with the spleen and transverse colon. Also evident is moderate ascites but no visible liver nodules.

**Fig. 5:**
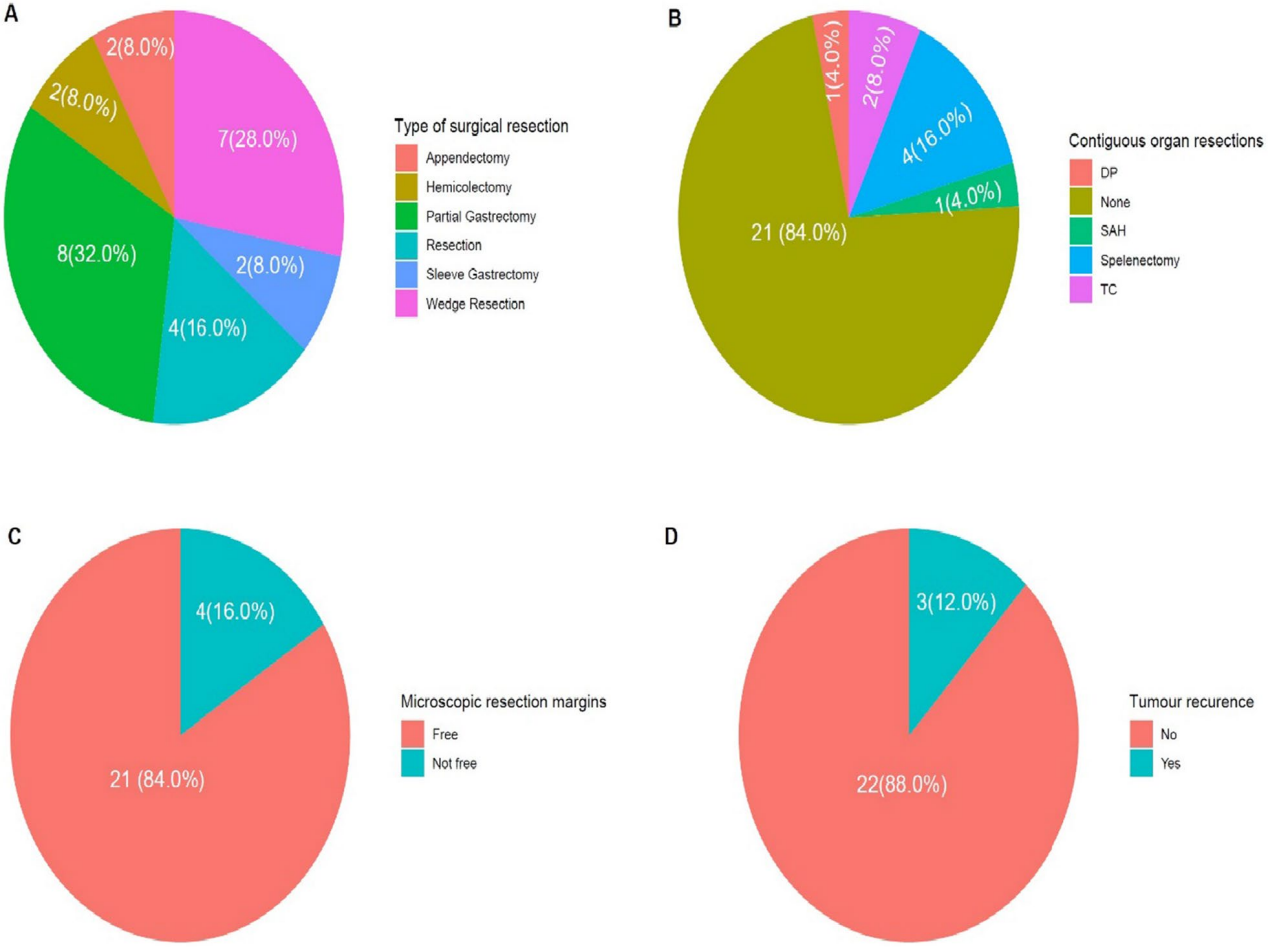
Frequency distribution of the surgical treatment options offered. *DP* distal pancreatectomy, *SAH* subtotal abdominal hysterectomy, *TC* transverse colectomy

Figure 5 shows the frequency distribution of the surgical treatment options offered. Majority of the subjects underwent Partial Gastrectomy (32.0%) while 7 subjects, representing 28.0% underwent Wedge Resection. Appendectomy and Sleeve Gastrectomy constituted the least performed procedure with frequencies of 8% each. Four out of the 25 patients (16.0%) had resections of involved contiguous organs done with Splenectomy, seen in all 4 patients being the most common procedure done as depicted in Fig. 5A, B. Free resection margins observed in 84% of the subjects while only 3/25 (12.0%) experienced tumour recurrence (Figs. 5C, D).

**Fig. 6:**
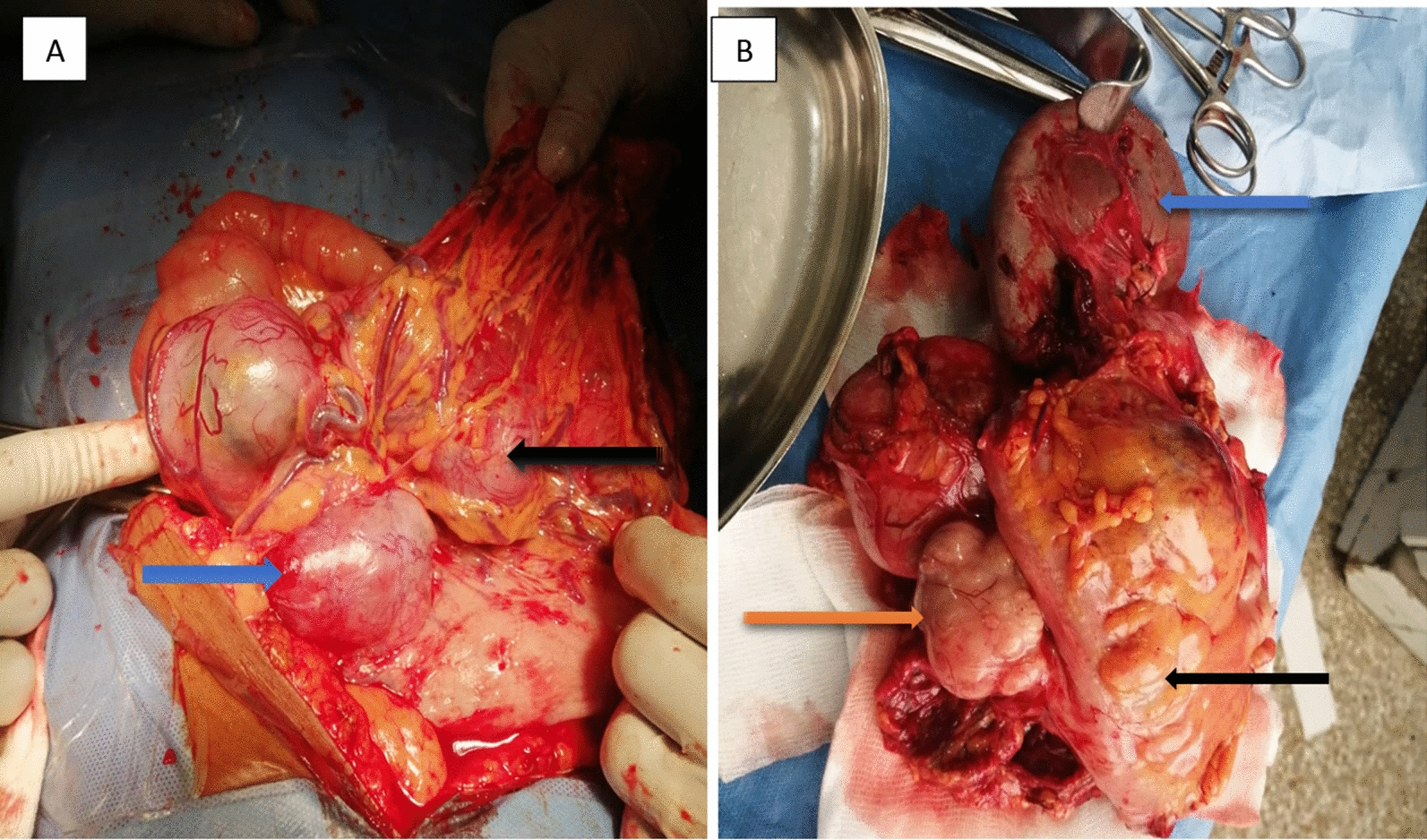
Intra-operative view of tumour (**A**) and pathologic specimen (**B**) of one patient

Figure 6 demonstrates a pathological specimen of one patient. The exophytic gastric tumour was found to be trapped in greater omentum (blue arrow—stomach; black arrow—tumour) as seen in Fig. 6A. Bloc excision of the mass (orange arrow) involved contiguous excision of the transverse colon (black arrow) and spleen (blue arrow) as demonstrated in Fig. 6B.

## Discussion

GIST has gained recognition as a distinct type, and the discovery of their origin from the interstitial cell of Cajal lineage in the 1990s over the years [2]. Since then, advances have been made that have substantially shaped the diagnostic work-up, refined subclassification and risk assessment, and improved patient clinical management. Currently, orally approved tyrosine kinase inhibitors targeting KIT and PDGFRA oncogenic activation have shown promising results [15]. Unfortunately, there is limited data on GIST patterns and clinical presentations in Ghana. In this study, we retrospectively reviewed all GIST cases from two tertiary hospitals in Ghana from January 2014 to June 2022.

Most GISTs occur sporadically among aged adults with a median age of 60 to 65 [1] though a minority can be found among children and young adults, where they may arise as part of the non-hereditary Carney triad [5]. The median age of subjects in this study was 50 years with most of them being above 61 years. This finding is consistent with reports from multiple studies, that have shown that GIST is more common in older patients, males, Blacks, and Asian/Pacific Islanders, with an average age at diagnosis ranging from 62 to 75 and a peak incidence in the 8th decade of life [3, 6, 12]. The demography of GIST patients in this study showed a female preponderance. This was in contrast to Van der Zwan et al. who reported a fairly consistent equal distribution between males and females [4]. A recent retrospective study in Nigeria by Agodirin et al. also showed a close ratio of 1:1 with a greater percentage being females [16].

Our results reveal that the stomach represented the most clinically presented tumour location followed by the appendix. Similar results have been reported by Patel et al. with a 68% incidence of GIST in the stomach while 12% of them were localized in the small bowel [17]. Extra-gastrointestinal stromal tumours (EGISTs) have been reported at sites including omentum, mesentery, retroperitoneum, gall bladder, pancreas, urinary bladder, and liver [6]. EGISTs characteristics are similar to GISTs with respect to histopathology and immunohistochemistry [7, 18]. We found one patient whose tumour was localized in the mesentery. The presence of GIST in these unexpected areas has been explained to be due to the accidental dispersion of interstitial cells of Cajal during the embryogenic period [19].

The clinical presentation of GISTs typically is dependent on the location of the primary tumour [18]. Up to about 20% of cases of GIST are asymptomatic due to their small size and as such usually diagnosed as incidental findings on computed tomography, endoscopy, or autopsy [4, 6, 17]. The presence of an abdominal mass (60%) was the most predominant symptom. Other rare presentations include dysphagia, hypoglycaemia, biliary obstruction and jaundice, and intussusception [17]. These observations were in line with other studies that found comparable results [12, 20]. Gastrointestinal bleeding has been reported as the most common symptom associated with GIST [6], a fact reiterated by Sorour et al. in Egypt where almost half of the 92 patients diagnosed with GIST presented with gastrointestinal bleeding [21]. Our study, however, demonstrated abdominal mass as the predominant symptom while gastrointestinal bleeding being seen in 40% of the study participants, similar to the 30–40% reported by Rammohan et al. in 2013 [22]. Bleeding associated with the tumour may be intraluminal, in which case they may present with hematemesis, melena, or even hematochezia [6, 23]. The differences in these symptoms may be due to variations in diagnosis and recording. Due to their exophytic pattern of growth, the mechanism of intestinal obstruction in GIST is usually due to extrinsic compression of the surrounding bowel by tumour rather than intraluminal overgrowth and occlusion. As such, intestinal obstruction in GIST is common in giant tumours [17]. A rare finding in our study showed only 3 out of the 25 patients had an intestinal obstruction. Similar findings have been published in a case report by Newme et al. in a tertiary hospital in India [24].

Metastasis most often occurs either by localized or hematogenous spread to the liver, omentum, and peritoneum while distant spread to the lungs, lymph nodes, and bones is rare [6, 17, 25]. Regarding metastasis formation, we observed peritoneal deposits and liver metastasis in 20% and 8% of patients respectively. The majority of the diagnoses of GIST are made after a histopathologic assessment of the resected mass [17]. Percutaneous biopsy of operable GIST tumours is not advisable due to the risk of intra-peritoneal seeding, tumour rupture, and haemorrhage. It is only reserved for inoperable or metastatic tumours or lesions in which the course of management may be altered if confirmed, as in lymphomas and germ cell tumours [4, 17, 25]. Macroscopically, they may present as small lesions measuring a few millimetres or centimetres, or giant masses over 30 cm in diameter with a median diameter of about 5–8 cm [6, 15]. Based on the microscopic morphology, they are classified as spindle cell, epithelioid, and mixed or pleomorphic types, with the spindle cell types being the most predominant (70%) [4, 15, 17]. On immunohistochemical analysis of GISTs, they demonstrate positivity to KIT receptor tyrosine kinase [15, 25]. The majority of them demonstrate either a c-kit or platelet-derived growth factor alpha (PDGRA) mutation [12]. Multiple studies have found DOG1 and CD117 as the most sensitive stains for confirmation of GISTs [8, 26].

Complete surgical resection in localized disease is deemed the gold standard of treatment as it is the only avenue that offers a potential cure [1, 14, 17, 27]. The surgeon should aim at the careful resection of the tumour while avoiding tumour rupture as it is associated with peritoneal seeding of the tumour and increased chances of peritoneal recurrence of the tumour [4, 15]. Several studies have re-echoed the insignificance of regional lymphadenectomy since lymph node metastasis in GIST is hardly seen [6, 28]. Due to the exophytic tendency of the tumour, en bloc resection of the tumour and contiguous structures may be warranted to achieve complete resection, in cases where it is found to be adherent to them [25, 27]. Splenectomy was the most frequent additional resection done in all four cases with contiguous organ resections having that procedure done. Overzealous resection of vital structures to achieve wider negative resection margins should be avoided since it may be associated with significant postoperative morbidity [3, 14]. Though a predominant finding in our study where 84% of the patients had tumour-free margins, recent studies have demonstrated that the achievement of microscopic tumour-free margins was not as vital an indicator for survival [10, 15].

GISTs usually have a high chance of recurrence despite complete surgical resection, with the most common sites for metastasis being the liver and the peritoneum. In the event of recurrence, the role of surgery is particularly limited, and chemotherapy also has proven to be an ineffective alternative [4, 6, 12].

In 2002, a risk stratification criterion for predicting the level of aggressiveness of GIST was proposed based on tumour size and mitotic index which classified them into very low, low, intermediate, and high risks [3]. This criterion, however, has since undergone modifications though high-risk GISTs have been documented to constitute 24–45% of GISTs [29]. Our study demonstrated a higher rate of 56% for high-risk GISTs and from our literature review, represent the first description of high-risk GISTs in Ghana. Imatinib, a tyrosine kinase inhibitor, has been approved worldwide following multiple trials for use in high-risk GISTs. It has been demonstrated to contribute to improved progression-free survival and recurrence-free survival when administered as an adjuvant treatment for about 3 years. Even though our study did not evaluate the outcomes among patients with GISTs, we suggest adjuvant administration of imatinib to our patients would demonstrate similar outcomes as documented in literature.

This study provides baseline evidence to highlight the gap in the literature regarding GIST in the Ghanaian population as well as provide valuable information on the Ghanaian picture for future reviews on the African perspective. However, it has some limitations including a retrospective design of the study, a small sample size of the study and a lack of data on follow-up time for survival analysis due to administrative challenges. For the purpose of diagnosis, treatment, and monitoring of GIST in Ghana as well as incorporating GIST into the population-based cancer registry established in 2012 in Ghana [28]. Thus, a large longitudinal multicentre study is warranted to provide a representative picture of the clinical and pathologic characteristics of GISTs as pertains among the Ghanaian population. This would provide answers on whether the tumour biology among our population is comparable to what pertains in other population.

## Conclusion

GIST is a potentially curable tumour that once was obscure but currently gaining popularity. It is more common among patients aged 60 years and above with abdominal mass and abdominal pain being the predominant clinical presentation. The stomach remains the primary location of occurrence with majority of tumours being high-risk GISTs. Surgical resection offers the hope of a cure for localized disease while Imatinib is a recommended adjuvant therapy for high-risk GISTs as well as a viable option for recurrent, metastatic, or unresectable tumours.

## Data Availability

The data presented in the results of this study will be made available on reasonable request to Dr. Tonnies Abeku Buckman. E-mail address: tonniesb@yahoo.com.

## References

[1] Casali P (2018). Gastrointestinal stromal tumours: ESMO–EURACAN clinical practice guidelines for diagnosis, treatment and follow-up. Ann Oncol.

[2] Cirocchi R (2014). Right hemicolectomy plus pancreaticoduodenectomy vs partial duodenectomy in treatment of locally advanced right colon cancer invading pancreas and/or only duodenum. Surg Oncol.

[3] Fletcher CD (2002). Diagnosis of gastrointestinal stromal tumors: a consensus approach. Hum Pathol.

[4] van der Zwan SM, DeMatteo RP (2005). Gastrointestinal stromal tumor: 5 years later. Cancer Interdiscip Int J Am Cancer Soc.

[5] Agaimy A, Wünsch PH (2006). Gastrointestinal stromal tumours: a regular origin in the muscularis propria, but an extremely diverse gross presentation. Langenbeck’s Arch Surg.

[6] Stamatakos M (2009). Gastrointestinal stromal tumor. World J Surg Oncol.

[7] Miettinen M (2003). Gastrointestinal stromal tumors, intramural leiomyomas, and leiomyosarcomas in the duodenum: a clinicopathologic, immunohistochemical, and molecular genetic study of 167 cases. Am J Surg Pathol.

[8] Caterino S (2011). Gastrointestinal stromal tumors: correlation between symptoms at presentation, tumor location and prognostic factors in 47 consecutive patients. World J Surg Oncol.

[9] Kapoor R (2013). Five-year follow up of patients with gastrointestinal stromal tumor: recurrence‐free survival by risk group. Asia‐Pac J Clin Oncol.

[10] Al-Kalaawy M (2012). Gastrointestinal stromal tumors (GISTs), 10-year experience: patterns of failure and prognostic factors for survival of 127 patients. J Egypt Natl Cancer Inst.

[11] Gutierrez JC (2007). Optimizing diagnosis, staging, and management of gastrointestinal stromal tumors. J Am Coll Surg.

[12] Sanchez-Hidalgo JM (2018). Gastrointestinal stromal tumors: a multidisciplinary challenge. World J Gastroenterol.

[13] Ahmed M (2020). Recent advances in the management of gastrointestinal stromal tumor. World J Clin Cases.

[14] Judson I (2017). UK clinical practice guidelines for the management of gastrointestinal stromal tumours (GIST). Clin Sarcoma Res.

[15] Joensuu H (2006). Gastrointestinal stromal tumor (GIST). Ann Oncol.

[16] Agodirin O (2020). Presentation intervals and the impact of delay on breast cancer progression in a Black African population. BMC Public Health.

[17] Patel N, Benipal B (2019). Incidence of gastrointestinal stromal tumors in the United States from 2001–2015: a United States cancer statistics analysis of 50 states. Cureus.

[18] Miettinen M, Lasota J (2006). Gastrointestinal stromal tumors: pathology and prognosis at different sites. Semin Diagn Pathol.

[19] Morgan J et al. Epidemiology, classification, clinical presentation, prognostic features, and diagnostic work-up of gastrointestinal stromal tumors (GIST). 2018. UpToDate.

[20] Novelli M (2010). DOG1 and CD117 are the antibodies of choice in the diagnosis of gastrointestinal stromal tumours. Histopathology.

[21] Sorour MA (2014). Gastrointestinal stromal tumors (GIST) related emergencies. Int J Surg.

[22] Rammohan A, Sathyanesan J, Rajendran K, Pitchaimuthu A, Perumal SK, Srinivasan UP, Ramasamy R, Palaniappan R, Govindan M (2013). A gist of gastrointestinal stromal tumors: a review. World J Gastrointest Oncol.

[23] Oluyemi A (2015). Gastrointestinal stromal tumor of the anal wall in a Nigerian. Pan Afr Med J.

[24] Newme K (2020). Acute intestinal obstruction due to gastrointestinal stromal tumours: case report. Int Surg J.

[25] Afuwape O, Irabor D, Ladipo J (2011). Gastrointestinal stromal tumour in Ibadan, Nigeria: a case report and review of current treatment. Afr Health Sci.

[26] Liu Q (2015). Study on clinicopathological features of gastrointestinal stromal tumor and relevant prognostic factors. Cell Biochem Biophys.

[27] Demetri GD (2007). NCCN Task Force report: management of patients with gastrointestinal stromal tumor (GIST)—update of the NCCN clinical practice guidelines. J Natl Compr Cancer Netw.

[28] Laryea DO (2014). Cancer incidence in Ghana, 2012: evidence from a population-based cancer registry. BMC Cancer.

[29] Huang H-Y (2007). A modification of NIH consensus criteria to better distinguish the highly lethal subset of primary localized gastrointestinal stromal tumors: a subdivision of the original high-risk group on the basis of outcome. Surgery.

